# The road to clinical use of high-intensity focused ultrasound for liver cancer: technical and clinical consensus

**DOI:** 10.1186/2050-5736-1-13

**Published:** 2013-08-01

**Authors:** Jean-Francois Aubry, Kim Butts Pauly, Chrit Moonen, Gail ter Haar, Mario Ries, Rares Salomir, Sham Sokka, Kevin Michael Sekins, Yerucham Shapira, Fangwei Ye, Heather Huff-Simonin, Matt Eames, Arik Hananel, Neal Kassell, Alessandro Napoli, Joo Ha Hwang, Feng Wu, Lian Zhang, Andreas Melzer, Young-sun Kim, Wladyslaw M Gedroyc

**Affiliations:** 1 Institut Langevin, ESPCI ParisTech, CNRS UMR 7587, INSERM U979, Université Denis Diderot, Paris VII, Paris, France; 2 Department of Radiation Oncology, University of Virginia, Charlottesville, VA, USA; 3 Radiological Sciences Laboratory, Stanford University School of Medicine, Palo Alto, CA, USA; 4 Imaging Division, University Medical Center Utrecht, Amsterdam, The Netherlands; 5 Division of Radiotherapy and Imaging, The Institute of Cancer Research, Royal Marsden Hospital, Sutton, Surrey, UK; 6 Faculty of Medicine, University of Geneva, Geneva, Switzerland; 7 Philips Healthcare, Boston, MA, USA; 8 Ultrasound Innovations Applications, Siemens Healthcare, Issaquah, WA, USA; 9 InSightec Ltd, 5 Nahum Heth Street, Haifa, Israel; 10 Chongqing Haifu Medical Technology Co., Ltd, Chongqing, China; 11 Focused Ultrasound Foundation, Charlottesville, VA, USA; 12 University of Rome, La Sapienza, Rome, Italy; 13 Digestive Disease Center, University of Washington, Seattle, WA, USA; 14 Institute of Ultrasonic Engineering in Medicine, Chongqing Medical University, Chongqing, China; 15 Clinical Center for Tumor Therapy, Second Affiliated Hospital of Chongqing University of Medical Sciences, Chongqing, China; 16 Institute for Medical Science and Technology, University of Dundee, Dundee, Scotland, UK; 17 Department of Radiology, Samsung Medical Center, Sungkyunkwan University School of Medicine, Seoul, South Korea; 18 Department of Medicine, Imperial College, South Kensington Campus, Exhibition Rd, London SW7 2AZ, UK; 19 Saint Mary’s Hospital, Praed St, W2 1NY, London, UK

**Keywords:** Ultrasound, Non-invasive surgery, High-intensity ultrasound, Focused ultrasound, Liver cancer

## Abstract

Clinical use of high-intensity focused ultrasound (HIFU) under ultrasound or MR guidance as a non-invasive method for treating tumors is rapidly increasing. Tens of thousands of patients have been treated for uterine fibroid, benign prostate hyperplasia, bone metastases, or prostate cancer. Despite the methods' clinical potential, the liver is a particularly challenging organ for HIFU treatment due to the combined effect of respiratory-induced liver motion, partial blocking by the rib cage, and high perfusion/flow. Several technical and clinical solutions have been developed by various groups during the past 15 years to compensate for these problems. A review of current unmet clinical needs is given here, as well as a consensus from a panel of experts about technical and clinical requirements for upcoming pilot and pivotal studies in order to accelerate the development and adoption of focused ultrasound for the treatment of primary and secondary liver cancer.

## Current overview and unmet clinical need for treating cancer in and of the liver

### Introduction

Although hepatocellular carcinoma and metastatic liver disease require completely separate analysis and study protocols, the technological approach of high-intensity focused ultrasound treatments is similar. Therefore, the two indications will be discussed in parallel as promising pilot studies. In addition, both available guidance methods (ultrasound-guided and MR-guided focused ultrasound) will be considered equally. Both approaches are applicable, and both have advantages and disadvantages.

### Primary liver tumors

The American Cancer Society's 2012 estimate for primary liver and bile duct cancers in the United States amounts to 28,720 new cases and 20,550 deaths [1]. Worldwide, liver cancer is the fifth most common cancer in men and the seventh in women. An estimated 748,300 new liver cancer cases occurred during 2008 [2]. Primary Liver cancer rates are the highest in East, Southeast Asia, West and Central Africa [2]. Primary liver cancer occurs most commonly in previously damaged livers, as in cases of viral hepatitis, alcohol abuse, obesity, and exposure to aflatoxin.

Treatment of liver cancer involves multiple strategies used independently, or in combination, depending on the stage of the disease. These include liver replacement therapy, local therapy (resection, ablation), and regional therapy. However, to date, only about 25% of the patients with primary liver tumors are considered to be suitable candidates for curative treatment. Reasons for patient non-eligibility for curative treatment include factors such as the underlying parenchymal disease state of the liver, tumor size, location, and multi-focality or multi-centricity. HIFU has the potential to improve these percentages by offering a local therapy which may be less limited in terms of patient selection and one that offers a lower threshold for treatment attempt in terms of patient morbidity and complication. For example the 2010 Treatment Guidelines for hepatocellular carcinoma from the American Association for the Study of Liver Disease [3] stated that radiofrequency ablation should be offered to patients with three or fewer primary tumors of less than 3 cm each, yet HIFU has the potential to target and ablate non-invasively, and in one session, a higher number of lesions with lower treatment-related morbidity.

Patients with more advanced disease are currently offered palliative treatment, including localized transarterial chemoembolization (TACE) or systemic chemotherapy. For this patient population, HIFU can offer the option to combine a low morbidity local therapy.

### Liver metastases

Metastatic liver tumors are more common than primary tumors in the USA. The most common sites of primary tumors that metastasize to the liver are breast, lung, and colon. Some authors have reported hepatic metastases in as many as 40% to 50% of adult patients with extra-hepatic primary tumors [4].

Colorectal cancer (CRC) is the second most commonly diagnosed cancer in Europe, with an annual incidence of 400,000 cases and an annual mortality of more than 200,000 patients [5]. Almost 70% of CRC patients develop liver metastases during the course of disease [6].

Curative treatments for patients with liver metastases can be aggressive in terms of the extent of damage to liver tissue since there is usually no underlying liver disease. The existence of a liver metastasis means that the primary cancer is stage IV and also requires systemic therapy, unless contraindicated by other patient factors.

For patients with CRC metastases, all treatment strategies are intended to cure [7] and are only limited by allowing sufficient parenchyma to remain for survival. Surgical resection can offer a 30%–50% 5-year and 17%–26% 10-year survival after a successful procedure [8,9]. Yet more than 80% of the patients are not surgical candidates because of insufficient residual liver tissue, extra-hepatic disease, anatomic constraints of the tumor, or medical comorbidities.

Without treatment, the median survival of patients with colorectal liver metastases is 6–12 months, and the 5-year survival is less than 10% [10]. Local ablative therapies targeting the liver metastases and surrounding tumor-free margins in patients without extra-hepatic metastases can achieve local tumor control and offer better survival [11,12], even in patients with more than four metastases [13] and in patients with repeated metastases [14,15]. Laser-induced thermo therapy, radiofrequency ablation, microwave ablation, cryoablation, and trans-vascular embolization have been applied for curative or palliative ablation of liver metastases [16]. Local ablative techniques result in a delayed and reduced residual intra-hepatic tumor growth and peritoneal tumor spread compared with hepatic resection [17] due to a reduced growth factor expression in comparison to surgical resection [18,19]. Also, for patients with liver metastases, HIFU has the potential to offer local therapy which may be less limited in terms of patient selection and one that theoretically should have lower patient morbidity and complication.

### Review of clinical trials and technological developments in ultrasonic liver therapy

#### Clinical trials

HIFU has been explored as a method for non-invasive localized thermal ablation for many years [20,21]. Vallancien et al. [22] was the first to report the use of therapeutic ultrasound to treat the liver. In this study, liver metastases were targeted under ultrasonic guidance with a retractable imaging probe in two patients. Imaging probes have more recently been integrated into the therapeutic heads on clinical devices: 474 patients with primary and metastatic liver cancer have been treated by Wu et al. [23] between 1997 and 2001 with the JC model from Chongqing Haifu (Chongqing, China). Other teams have taken advantage of magnetic resonance (MR) imaging to monitor liver tumor treatments [24,25]. Nevertheless, the ultrasound (US)-guided JC model remains to date the only system with a significant clinical track record in liver and a regulatory approval for liver applications (CE mark and Chinese CFDA).

All these clinical studies suggest that HIFU may be a safe and feasible technique capable of complete tumor ablation, but the same reports describe a significant incidence of skin burns. [23,26-28]. The presence of the rib cage enhances the probability of skin overheating due to the high value of the ultrasonic absorption coefficient of the bone [29] which causes indirect skin heating. In vivo measurements of temperature elevation on pork ribs have been reported by Daum et al. [30] with MR temperature monitoring: temperature elevation during sonication was five times higher on the ribs than in the intercostal space. Moreover, reflection and refraction of the ultrasonic wave by the rib cage affects the focusing [31] and, thus, degrades the efficiency of the treatment at the target.

### Technological developments

#### Shadowing effect of the ribs

In order to minimize the heating effects of the ribs, most of the clinical studies cited previously have taken advantage of sub-costal sonication. Wu et al. [26] proposed the resection of a portion of the ribs overlying the targeted region of the liver in order to provide an acoustic window. It was suggested in the late 1990s that the liver could be treated using a phased array to sonicate between the ribs [32-34], but this could not be tested experimentally at that time. Civale et al. [35] recently lowered the temperature of the ribs during sonication by switching off up to three elements of a linearly segmented HIFU transducer. Even though no focal lesions were induced and the energy from the remaining active elements was not sufficient for a clinical application, this study confirmed the proof of concept introduced by McGough et al. [32] and Botros et al. [33]. Multi-element-phased arrays made of hundreds of elements have since been developed [36-39]. Aubry et al. [40] used a 300-element semi-random array to show that time reversal is well suited in focusing through the rib cage for therapeutic applications: the temperature elevation on the rib surface was decreased to a negligible level (a mean of 0.3°C). This can be achieved automatically and non-invasively by computing the decomposition of the time reversal operator based on the backscattered echoes [41,42]. Such a technique makes it possible to focus between the ribs and not through the ribs. This can also be achieved by physical blocking using a mask [43].

#### Breathing motion

Another medical and technical characteristic of ultrasonic treatment of the liver is that respiratory movement affects the precision and the efficiency of all extracorporeal treatments—HIFU as well as radiotherapy. Several studies [44-47] have shown that abdominal organs can move up to 20 mm during a respiratory cycle, reaching speeds of up to 15 mm/s.

(a) Breath holding: One solution is to have the patient undergo ventilator-controlled breath-holds while under general anesthesia. This has been used effectively in small patient studies [48,49].

(b) MR-based motion tracking: Alternatively, a technologically more difficult solution is to steer the beam during continuous breathing. This requires tissue-motion tracking, and a variety of methods have been investigated for tracking liver motion in the MR system. Navigator echoes have been investigated [50]. de Senneville et al. [51] proposed a technique based on an analytical model of the main global motion defined during a pretreatment procedure. The average motion is then estimated and used in order to anticipate the motion during treatment. Ries et al. [52] proposed tracking the target position in the image plane with 2D optical flow-based image registration, while out-of-plane motion is compensated by dynamic slice tracking. This technique allows sufficient temporal resolution and precision but is very sensitive to the tracking frequency and beam steering latency.

(c) US-based motion tracking: For US-guided HIFU, ultrasound-based techniques have been shown to be able to track the 3D motion of biological tissues locally [53-56]. Such an approach is based on tracking temporal shifts in the backscattered RF signals, resulting from the displacements of the tissues. The main advantage of the ultrasound-based method is the high penetration rate of ultrasound in the human body and its real-time capabilities. Hence, the natural ultrasonic scatterers in biological tissue can be used as markers to track the local motion of tissues located deep within organs.

(d) Motion compensation by electronic beam steering: Once the 3D movement of the organ is measured, the ultrasonic beam can be electronically steered in order to compensate for this and to follow the tissue motion in real time. Marquet et al. [57] achieved motion compensation ten times per second by interleaving ultrasonic motion detection during the first 20 ms, followed by electronic beam steering calculation and hardware phase adjustment (10 ms) and 70-ms high-intensity sonication, allowing a 70% duty cycle while tracking the organ. Two studies pulled together the required technologies of motion tracking, beam steering, and MR thermometry to demonstrate the production of HIFU lesions during continuous breathing [58,59].

### Monitoring

(a) MR temperature monitoring in the presence of motion: In order to work, the MR thermometry method must be insensitive to respiratory motion [60]. This requires that both the thermometry processing method and the acquired data are robust in the presence of such movement. For the processing method, multi-baseline [50,51,61,62], referenceless [63-65] and hybrid multi-baseline/referenceless [66] techniques have been developed. With these techniques available in processing the thermometry data, the acquired data has been shown to be robust in the presence of respiratory-like motion in a phantom [67] and a pig model [58,59].

(b) US monitoring in the presence of motion: Most clinical studies in liver have been performed with B-mode ultrasonic monitoring [23,26-28] of a hyperechogenic signature of the treated area. A rate of 20 to 50 frames per second can be achieved, so that such monitoring is not affected by the liver motion. Nevertheless, liver motion has a major impact on US-based temperature monitoring as this relies on tracking apparent displacements due to the heat-related change of the speed of sound [68,69]. Such apparent displacement and liver motion are superimposed, making it difficult to differentiate between each effect. Elastography-based temperature monitoring [70] might be the most practical way of achieving US temperature monitoring in the liver.

### Unmet needs

The current unmet need is to provide non-invasive, low morbidity, localized tissue destruction to the significant patient population that is currently being offered palliative treatment. HIFU should be able to provide this due to its non-invasive and highly precise nature. HIFU can be used in close proximity to sensitive structures, and it has the potential to target multiple lesions without the need for multiple incisions or needle insertions (which may cause bleeding, infection, and/or tumor spread). Focused ultrasound also has the potential to facilitate focal targeted drug release which in the future may be an option to enhance chemotherapy.

### Pilot study consensus

A pilot study should address the feasibility and safety of localized tissue destruction using HIFU and provide the information needed to design a pivotal study. The recommendation of the assembled quorum is to choose patients with localized liver lesions who are scheduled for surgery and to treat these patients using HIFU before the surgery, (i.e., ablate-and-resect study). This approach can provide the following outcomes:

(1) Short-term safety: All patients would be followed for 1 week following their HIFU ablation procedure to record any immediate post-procedural adverse effects (such as bleeding, damage to bowel or adjacent organs, and near-field skin/fat/muscle burns).

(2) Post-treatment radiological follow-up data: Following treatment, patients would be imaged by contrast-enhanced MR imaging and/or computed tomography (CT or PET CT if available) to record the post-treatment appearance of the treated lesion, to correlate it with pathological findings, and to asses targeting accuracy.

(3) Post-treatment pathological findings: The excised specimen would be used as a gold standard in an analysis which evaluates correlation between the planned, imaged, and ablated tissue, and would confirm the completeness of the tissue destruction.

Since the goal of the pilot study is to establish evidence for the efficacy and safety of HIFU for localized thermal ablation of tissue in the liver, the patient population could include both primary and secondary liver tumor patients. However, it would be advisable that the first several patients recruited and treated in this study would be CRC patients in order to reduce the risk of introducing patient/liver-related complications due to the underlying liver disease in primary liver tumor patients.

The proposed pilot study is a single-arm ablate-and-resect study, targeting patients with primary or secondary liver cancer who are candidates for surgical resection. After enrollment, the patients would undergo HIFU treatment and be followed for a period of 1 week. After 1 week, the treated lesion would be excised and sent for pathological analyses. A potential approach to gradually increasing total dose used could be in the initial treatments to partially ablate the targeted tumor tissue and (2) in later treatments to completely ablate the tumors, including a tumor margin.

### Pivotal study

The pivotal study should target the patient population identified in the unmet need section: primary liver cancer patients who are not candidates for surgical resection and who are receiving palliative treatment. This patient population could include patients with more than three lesions, patients with one lesion bigger than 5 cm, patients with ascites, or patients with lesions close to major blood vessels or other structures that make resection or radiofrequency ablation difficult or impossible. The specifics of what population to include from the list above would take into consideration results and learned lessons on HIFU capabilities in terms of accuracy and treatment speed as were demonstrated in the pilot study. We suggest a double-arm study where the control arm would get TACE and the test arm would get TACE and HIFU. The sample size would be determined by existing evidence in the literature regarding the efficacy of such combination treatment [71].

The rationale for the study is the need to avoid the risk of withholding existing therapy from a severely ill patient population, while considering the near-term reality of where to maximize clinical benefit; it is expected that these patients would receive combination therapy anyway.

An acceptable procedure, to be done in preparation for the HIFU ablation session, is to insert saline or another fluid into the peritoneal cavity to move the bowels away from the targeted area or into the pleural cavity to move the lungs away from the acoustic pathway. This procedure can be done if necessary for safety, but should not be part of the routine.

Follow-up would include imaging, measurement of alpha fetoprotein levels (although effectiveness may be compromised due to TACE), liver function testing, and record of disease-related events including local, regional and distant recurrences, disease-specific survival, disease-free survival, and overall survival.

As a future second step, it is envisioned that a three-arm study comparing the safety and efficacy of TACE alone, HIFU alone, and the combination of HIFU and TACE will be conducted. This second, three-arm study would be needed to obtain regulatory approval for the usage of HIFU ablation of primary liver tumor as a stand-alone therapy. A separate pivotal trial for the metastatic liver disease indication would be required.

### Minimal technical specifications of a HIFU system for the treatment of liver tumors

The many aspects and technological improvements in HIFU technology, which are required to treat liver cancer have been researched for years (‘Technological developments’ section). However, as part of our recommendation, we tried to narrow the technical specifications to the minimum required for a future or existing HIFU system to be commercially viable in terms of patient selection, quality and cost of treatment, and technological availability and cost. We have identified the following minimal requirements:

● Treatment rate should be at least 1 cc of ablated tissue per minute.

● The entire procedure should last 4 hours or less (and has a potential of reducing time to 2 hours).

● The system should be able to target at least 80% of the liver volume in a typical patient.

● The system should be able to transmit energy either in between, below, or through the ribs without damaging the ribs or causing a skin burn.

● The system should have an imaging guidance system for targeting tumors.

● It would be beneficial to have closed loop thermometry (MR-based or otherwise)

● It would be beneficial to have beam steering tissue tracking to enable the patient to breathe freely, but this is not a ‘must have’ feature, and carrying out treatment with the patient under general anesthesia using forced apnea is acceptable.

Each of these components has been developed separately or by academic research groups.

## Competing interests

JFA, KBP, GtH, RS, AN, JHH, FW, LZ, YK, and WMG declare no competing interests. CM and MR acknowledge research collaboration with Philips Healthcare. SS is currently employed by Philips Healthcare. KMS is currently employed by Siemens Healthcare. YS is currently employed by InSightec. FY is currently employed by Chongqing HAIFU. HHS and ME are currently employed by the Focused Ultrasound Foundation. AH and NK are currently employed by the Focused Ultrasound Foundation and share holders at InSightec. AM acknowledges research collaboration with GE and InSightec and funding by European Union.

## Authors’ contributions

All the authors participated in at least one of the two workshops organized by the focused ultrasound foundation to establish this consensus. The minutes of the meetings were collected by ME. First draft was written by AH and JFA. Active editing and commenting were done by YSK, LZ, AM, WMG, CM, KBP, GTH, RS, KMS, and SS. All authors read and approved the final manuscript.

## References

[B1] American Cancer Society Cancer facts and figures 2012 2012 Atlanta, GA: American Cancer Society [cited 2012 September 6]. Available from: http://www.cancer.org/Research/CancerFactsFigures/CancerFactsFigures/cancer-facts-figures-2012

[B2] American Cancer Society Global cancer facts and figures 2012 Atlanta, GA: 2nd edition. American Cancer Society [cited 2012 September 6]. Available from: http://www.cancer.org/Research/CancerFactsFigures/GlobalCancerFactsFigures/global-facts-figures-2nd-ed

[B3] Bruix J Sherman M Management of hepatocellular carcinoma: an update 2010 Alexandria (VA, USA): American Association for the Study of Liver Diseases [cited 2012 September 6]. Available from: http://guidelines.gov/content.aspx?id=23927 10.1002/hep.24199 PMC3084991 21374666

[B4] Ananthakrishnan A Gogineni V Saeian K Epidemiology of primary and secondary liver cancers Semin Intervent Radiol 2006 23 1 47 63 10.1055/s-2006-939841 21326720 PMC3036307

[B5] Ferlay J Autier P Boniol M Heanue M Colombet M Boyle P Estimates of the cancer incidence and mortality in Europe in 2006 Ann Oncol 2007 18 581 92 17287242 10.1093/annonc/mdl498

[B6] Ruers T Bleichrodt RP Treatment of liver metastases, an update on the possibilities and results Eur J Cancer 2002 38 1023 33 10.1016/S0959-8049(02)00059-X 11978527

[B7] Vauthey JN Choti MA Helton WS AHPBA/SSO/SSAT Consensus Conference on hepatic colorectal metastases: rationale and overview of the conference. January 25, 2006 Ann Surg Oncol 2006 13 10 1259 60 10.1245/s10434-006-9017-9 16952025

[B8] O'Rourke AP Haemerich D Prakash P Converse MC Mahvi DM Webster JG Current status of liver ablation devices Expert Rev Med Devices 2007 4 523 37 10.1586/17434440.4.4.523 17605688

[B9] Kopetz S Chang GJ Overman MJ Improved survival in metastatic colorectal cancer is associated with adoption of hepatic resection and improved chemotherapy J Clin Oncol 2009 27 3677 83 10.1200/JCO.2008.20.5278 19470929 PMC2720081

[B10] Bengmark S Hafstrom L The natural history of primary and secondary malignant tumors of the liver I 1969 23 198 202 10.1002/1097-0142(196901)23:1<198::aid-cncr2820230126>3.0.co;2-j 5763253

[B11] Konopke R Roth J Volk A Pistorius S Folprecht G Zöphel K Schuetze C Laniado M Saeger HD Kersting S Colorectal liver metastases: an update on palliative treatment options J Gastrointestin Liver Dis 2012 21 83 91 22457864

[B12] Vogl TJ Jost A Nour-Eldin NA Mack MG Zangos S Naguib NN Repeated transarterial chemoembolisation by different chemotherapeutic drug combinations followed by MR-guided laser-induced thermotherapy in patients with liver metastases of colorectal carcinoma Br J Cancer 2012 106 7 1274 79 10.1038/bjc.2012.69 22382689 PMC3314788

[B13] Minagawa M Makuuchi M Torzilli G Takayama T Kawasaki S Kosuge T Yamamoto J Imamura H Extension of the frontiers of surgical indications in the treatment of liver metastases from colorectal cancer: long-term results Ann Surg 2000 231 487 99 10.1097/00000658-200004000-00006 10749608 PMC1421023

[B14] Nagakura S Shirai Y Suda T Hatakeyama K Multiple repeat resections of intra- and extrahepatic recurrences in patients undergoing initial hepatectomy for colorectal carcinoma metastases World J Surg 2002 26 141 47 10.1007/s00268-001-0196-z 11865339

[B15] Petrowsky H Gonen M Jarnagin W Lorenz M DeMatteo R Heinrich S Encke A Blumgart L Fong Y Second liver resections are safe and effective treatment for recurrent hepatic metastases from colorectal cancer: a bi-institutional analysis Ann Surg 2002 235 863 71 10.1097/00000658-200206000-00015 12035044 PMC1422517

[B16] Goldberg SN Grassi CJ Cardella JF Charboneau JW Dodd GD III Dupuy DE Gervais DA Gillams AR Kane RA Lee FT Jr Livraghi T McGahan J Phillips DA Rhim H Silverman SG Solbiati L Vogl TJ Wood BJ Vedantham S Sacks D Society of Interventional Radiology Technology Assessment Committee and the International Working Group on Image-guided Tumor Ablation Image-guided tumor ablation: standardization of terminology and reporting criteria J Vasc Interv Radiol 2009 20 Suppl 7 S377 90 19560026 10.1016/j.jvir.2009.04.011

[B17] Isbert C Boerner A Ritz JP Schuppan D Buhr HJ Germer CT In situ ablation of experimental liver metastases delays and reduces residual intrahepatic tumour growth and peritoneal tumour spread compared with hepatic resection Br J Surg 2002 89 10 1252 59 10.1046/j.1365-2168.2002.02205.x 12296892

[B18] Slooter GD Marquet RL Jeekel J Ijzermans JN Tumour growth stimulation after partial hepatectomy can be reduced by treatment with tumour necrosis factor alpha Br J Surg 1995 82 1 129 32 10.1002/bjs.1800820144 7881931

[B19] Meredith K Haemmerich D Qi C Mahvi D Hepatic resection but not radiofrequency ablation results in tumor growth and increased growth factor expression Ann Surg 2007 245 5 771 76 10.1097/01.sla.0000261319.51744.59 17457170 PMC1877067

[B20] Ter Haar G Coussios C High intensity focused ultrasound: physical principles and devices Int J Hyperthermia 2007 23 2 89 104 10.1080/02656730601186138 17578335

[B21] Orsi F Arnone P Chen W Zhang L High intensity focused ultrasound ablation: therapeutic option for solid tumors J Cancer Res Ther 2010 4 414 20 21358073 10.4103/0973-1482.77064

[B22] Vallancien G Harouni M Veillon B Mombet A Praponitch D Brisset JM Bougaran J Focused extracorporeal pyrotherapy: feasibility study in man J Endourol 1992 6 2 173 81 10.1089/end.1992.6.173

[B23] Wu F Wang ZB Chen WZ Extracorporeal high intensity focused ultrasound ablation in the treatment of 1038 patients with solid carcinomas in China: an overview Ultrason Sonochem 2004 11 3–4 149 54 15081972 10.1016/j.ultsonch.2004.01.011

[B24] Okada A Murakami T Mikami K Onishi H Tanigawa N Marukawa T Nakamura H A case of hepatocellular carcinoma treated by MR-guided focused ultrasound ablation with respiratory gating Magn Reson Med Sci 2006 5 3 167 71 10.2463/mrms.5.167 17139143

[B25] Kopelman D Inbar Y Hananel A Freundlich D Castel D Perel A Greenfeld A Salamon T Sareli M Valeanu A Papa M Magnetic resonance-guided focused ultrasound surgery (MRgFUS): ablation of liver tissue in a porcine model Eur J Radiol 2006 59 157 62 10.1016/j.ejrad.2006.04.008 16725294

[B26] Wu F Zhi-Biao W Wen-Zhi C Hui Z Jin B Jian-Zhong Z Ke-Quan L Cheng-Bing J Fang-Lin X Hai-Bing S Extracorporeal high intensity focused ultrasound ablation in the treatment of patients with large hepatocellular carcinoma Ann Surg Oncol 2004 11 1061 69 10.1245/ASO.2004.02.026 15545506

[B27] Kennedy JE Wu F ter Haar GR High-intensity focused ultrasound for the treatment of liver tumours Ultrasonics 2004 42 931 35 10.1016/j.ultras.2004.01.089 15047409

[B28] Li JJ Xu GL Gu MF Complications of high intensity focused ultrasound in patients with recurrent and metastatic abdominal tumors World J Gastroenterol 2007 13 19 2747 51 17569147 10.3748/wjg.v13.i19.2747 PMC4147127

[B29] Goss SA Frizzell LA Dunn F Ultrasonic absorption and attenuation in mammalian tissues Ultrasound Med Biol 1979 5 2 181 86 10.1016/0301-5629(79)90086-3 556199

[B30] Daum DR Smith NB King R Hynynen K In vivo demonstration of noninvasive thermal surgery of the liver and kidney using an ultrasonic phased array Ultrasound Med Biol 1999 25 7 1087 98 10.1016/S0301-5629(99)00053-8 10574341

[B31] Kennedy JE Clarke RL ter Haar GR The effects of absorbers such as ribs in the HIFU beam-path on the focal profile Second International Symposium on Therapeutic Ultrasound 2002 185 92

[B32] McGough RJ Kessler ML Ebbini ES Cain CA Treatment planning for hyperthermia with ultrasound phased arrays IEEE Trans Ultrason Ferroelectr Freq Contr 1996 43 1074 84

[B33] Botros YY Volakis JL VanBaren P Ebbini ES A hybrid computational model for ultrasound phased-array heating in presence of strongly scattering obstacles IEEE Trans Biomed Eng 1997 44 1039 50 10.1109/10.641331 9353983 PMC2844506

[B34] Botros YY Ebbini ES Volakis JL Two-step hybrid virtual array-ray (VAR) technique for focusing through the rib cage IEEE Trans Ultrason Ferroelect Freq Contr 1998 45 989 99 10.1109/58.710577 18244253

[B35] Civale J Clarke R Rivens I ter Haar G The use of a segmented transducer for rib sparing in HIFU treatments Ultrasound Med Biol 2006 32 11 1753 61 10.1016/j.ultrasmedbio.2006.06.005 17112961

[B36] Daum DR Buchanan MT Fjield T Hynynen K Design and evaluation of a feedback based phased array system for ultrasound surgery IEEE Trans Ultrason Ferroelectr Freq Contr 1998 45 43 8 10.1109/58.660153 18244194

[B37] Chapelon JY Cathignol D Cain C Ebbini E Kluiwstra JU Sapozhikov O Fleury G Berriet R Chupin L Guey JL New piezoelectric transducers for therapeutic ultrasound Ultrasound Med Biol 2000 26 1 153 59 10.1016/S0301-5629(99)00120-9 10687803

[B38] Pernot M Aubry JF Tanter M Thomas JL Fink M High power transcranial beam steering for ultrasonic brain therapy Phys Med Biol 2003 48 16 2577 89 10.1088/0031-9155/48/16/301 12974575 PMC3002099

[B39] Ebbini ES Yao H Shrestha A Dual-mode ultrasound phased arrays for image-guided surgery Ultrasonic Imaging 2006 28 2 65 82 10.1177/016173460602800201 17094688

[B40] Aubry JF Pernot M Marquet F Tanter M Fink M Transcostal high–intensity-focused ultrasound: ex vivo adaptive focusing feasibility study Phys Med Biol 2008 53 2937 51 10.1088/0031-9155/53/11/012 18475006 PMC3021953

[B41] Cochard E Prada C Aubry JF Fink M Ultrasonic focusing through the ribs using the DORT method Med Phys 2009 36 8 3495 503 10.1118/1.3159755 19746783

[B42] Cochard E Aubry JF Tanter M Prada C Adaptive projection method applied to three-dimensional ultrasonic focusing and steering through the ribs J Acoust Soc Am 2011 130 2 716 23 10.1121/1.3607419 21877786

[B43] Salomir R Petrusca L Auboiroux V Muller A Vargas MI Morel DR Goget T Breguet R Terraz S Hopple J Montet X Becker CD Viallon M Magnetic resonance-guided shielding of prefocal acoustic obstacles in focused ultrasound therapy: application to intercostal ablation in liver Invest Radiol 2013 48 6 366 80 10.1097/RLI.0b013e31827a90d7 23344514

[B44] Bryan PJ Custar S Haaga JR Balsara V Respiratory movement of the pancreas: an ultrasonic study J Ultra Med 1984 3 317 20 10.7863/jum.1984.3.7.317 6748150

[B45] Ross CS Hussey DH Pennington EC Stanford W Doombos JF Analysis of movement of intrathoracic neoplasms using ultrafast computerized tomography Int J Radiat Oncol Biol Phys 1990 18 671 77 10.1016/0360-3016(90)90076-V 2318701

[B46] Korin HW Ehman RL Riederer SJ Felmlee JP Grimm RC Respiratory kinematics of the upper abdominal organs: a quantitative study Magn Reson Med 1992 23 1 172 78 10.1002/mrm.1910230118 1531152

[B47] Davies SC Hill AL Holmes RB Halliwell M Jackson PC Ultrasound quantitation of respiratory organ motion in the upper abdomen Br J Radiol 1994 67 1096 102 10.1259/0007-1285-67-803-1096 7820402

[B48] Visioli AG Rivens IH ter Haar GR Horwich A Huddart RA Moskovic E Padhani A Glees J Preliminary report of a phase I dose escalation study using focused ultrasound in the treatment of localised tumours Eu J Ultrasound 1999 9 11 8 10.1016/S0929-8266(99)00009-9 10099162

[B49] Gedroyc WM New clinical applications of magnetic resonance-guided focused ultrasound Top Magn Reson Imaging 2006 17 3 189 94 10.1097/RMR.0b013e318038f782 17414076

[B50] Vigen KK Daniel BL Pauly JM Butts K Triggered, navigated, multi-baseline method for proton resonance frequency temperature mapping with respiratory motion Magn Reson Med 2003 50 5 1003 10 10.1002/mrm.10608 14587011

[B51] de Senneville BD Mougenot C Moonen CT Real-time adaptive methods for treatment of mobile organs by MRI-controlled high-intensity focused ultrasound Magn Reson Med 2007 57 319 30 10.1002/mrm.21124 17260361

[B52] Ries M de Senneville BD Roujol S Berber Y Quesson B Moonen CT Real-time 3D target tracking in MRI guided focused ultrasound ablations in moving tissues Magn Reson Med 2010 64 1704 12 10.1002/mrm.22548 20878763

[B53] Hsu A Miller NR Evans PM Bamber JC Webb S Feasibility of using ultrasound for real-time tracking during radiotherapy Med Phys 2005 32 1500 14 10.1118/1.1915934 16013706

[B54] Tanter M Pernot M Aubry JF Montaldo G Marquet F Fink M Compensating for bone interfaces and respiratory motion in high intensity focused ultrasound Int J Hyperthermia 2007 23 2 141 51 10.1080/02656730701209996 17578338

[B55] Harris EJ Miler NR Bamber JC Evans PM Symonds-Tayler JRN Performance of ultrasound based measurements of 3D displacement using a curvilinear probe for organ motion tracking Phys Med Biol 2007 52 5683 703 10.1088/0031-9155/52/18/014 17804889

[B56] Schwartz BM McDannold NJ Ultrasound echoes as biometric navigators Magn Reson Med 2013 69 4 1023 33 10.1002/mrm.24336 22648783 PMC4099472

[B57] Marquet F Aubry JF Pernot M Fink M Tanter M Optimal transcostal high-intensity focused ultrasound with combined real-time 3D movement tracking and correction Phys Med Biol 2011 56 22 7061 80 10.1088/0031-9155/56/22/005 22016152

[B58] Quesson B Laurent C Maclair G de Senneville BD Mougenot C Ries M Carteret T Rullier A Moonen CT Real-time volumetric MRI thermometry of focused ultrasound ablation in vivo: a feasibility study in pig liver and kidney NMR Biomed 2011 24 2 145 53 10.1002/nbm.1563 21344531

[B59] Holbrook AB Ghanouni P Santos JM Dumoulin C Medan Y Butts Pauly K Respiration based steering for high intensity focused ultrasound liver ablation Magn Reson Med In press 10.1002/mrm.24695 PMC4040338 23460510

[B60] De Senneville BD Ries M Bartels LW Moonen CT In Interventional Magnetic Resonance Imaging MRI-guided high-intensity focused ultrasound sonication of liver and kidney 2012 New York: Springer: Edited by Kahn T, Busse H 349 66

[B61] Vigen KK Jarrard J Rieke V Frisoli J Daniel BL Butts PK In vivo porcine liver radiofrequency ablation with simultaneous MR temperature imaging J Magn Reson Imaging 2006 23 4 578 84 10.1002/jmri.20528 16508928

[B62] Shmatukha AV Bakker CJ Correction of proton resonance frequency shift temperature maps for magnetic field disturbances caused by breathing Phys Med Biol 2006 51 18 4689 705 10.1088/0031-9155/51/18/015 16953050

[B63] Rieke V Vigen KK Sommer G Daniel BL Pauly JM Butts K Referenceless PRF shift thermometry Magn Reson Med 2004 51 6 1223 31 10.1002/mrm.20090 15170843

[B64] Kuroda K Kokuryo D Kumamoto E Suzuki K Matsuoka Y Keserci B Optimization of self-reference thermometry using complex field estimation Magn Reson Med 2006 56 4 835 43 A published erratum appears in *Magn Reson Med* 2007, 57(4):812 10.1002/mrm.21016 16944467

[B65] Salomir R Viallon M Kickhefel A Roland J Morel DR Petrusca L Auboiroux V Goget T Terraz S Becker CD Gross P Reference-free PRFS MR-thermometry using near-harmonic 2-D reconstruction of the background phase IEEE Trans Med Imaging 2012 31 2 287 301 21937345 10.1109/TMI.2011.2168421

[B66] Grissom WA Rieke V Holbrook AB Medan Y Lustig M Santos J McConnell MV Pauly KB Hybrid referenceless and multibaseline subtraction MR thermometry for monitoring thermal therapies in moving organs Med Phys 2010 37 9 5014 26 10.1118/1.3475943 20964221 PMC2945742

[B67] Holbrook AB Santos JM Kaye E Rieke V Pauly KB Real-time MR thermometry for monitoring HIFU ablations of the liver Magn Reson Med 2010 63 2 365 73 10.1002/mrm.22206 19950255 PMC3212435

[B68] Pernot M Tanter M Bercoff KR Waters M Fink Temperature estimation using ultrasonic spatial compound imaging IEEE Ultras Ferr Freq Contr 2004 51 5 15217237

[B69] Liu D Ebbini ES Real-time 2-D temperature imaging using ultrasound IEEE Trans Biomed Eng 2010 57 1 12 6 10.1109/TBME.2009.2035103 PMC2843819 19884075

[B70] Sapin-de Brosses E Pernot M Tanter M The link between tissue elasticity and thermal dose in vivo Phys Med Biol 2011 56 24 7755 65 10.1088/0031-9155/56/24/005 22094357

[B71] Wu F Wang ZB Chen WZ Zou JZ Bai J Zhu H Li KQ Jin CB Xie FL Su HB Advanced hepatocellular carcinoma: treatment with high-intensity focused ultrasound ablation combined with transcatheter arterial embolization Radiology 2005 235 2 659 67 10.1148/radiol.2352030916 15858105

